# Can Total Wrist Arthroplasty Be an Option for Treatment of Highly Comminuted Distal Radius Fracture in Selected Patients? Preliminary Experience with Two Cases

**DOI:** 10.1155/2015/380935

**Published:** 2015-09-29

**Authors:** Ingo Schmidt

**Affiliations:** Hospital Schleiz GmbH, Department of Hand Surgery, Berthold-Schmidt-Straße 7-9, 07907 Schleiz, Germany

## Abstract

We present two case reports of successful primary shortening of the forearm and total wrist arthroplasty (TWA) using the new angle-stable Maestro Wrist Reconstructive System (WRS) for treatment of highly comminuted distal radius fracture in selected autonomous patients. In a 56-year-old male patient with adequate bone stock, insertion of the noncemented Maestro WRS was combined with ulnar shortening osteotomy. In an 84-year-old female patient with poor osteoporotic bone stock, insertion of the radial cemented Maestro WRS was combined with ulnar head resection. Both patients could resume their work without additional surgery after TWA. At the 1-year follow-up, there were no changes in position of either implant without signs of loosening, no impingement, and no instability of the distal radioulnar joint or the distal ulna stump. All clinical parameters (DASH score, pain through VAS, and grip strength) were satisfactory. Both patients reported that they would have the same procedure again. Further experience is needed to validate this concept.

## 1. Introduction and Technical Note

Distal radius fracture (DRF) is the most common fracture of the upper extremity, representing 16% of all fractures treated in emergency departments [1]. Primary surgical options would include internal locked volar or dorsal plating, joint bridging, or nonbridging external fixation with or without percutaneous pinning using* Kirschner-* (K-) wires, sole percutaneous pinning, and internal distraction plating. However, all of these techniques have drawbacks [2–7]. The primary wrist hemiarthroplasty with or without replacement of distal radius metaphysis for treatment of highly comminuted DRFs in elderly patients may help avoid secondary procedures related to posttraumatic wrist joint osteoarthritis (OA) and can lead to a faster restoration of their ability to work and independence [8–11].

**Figure 1:**
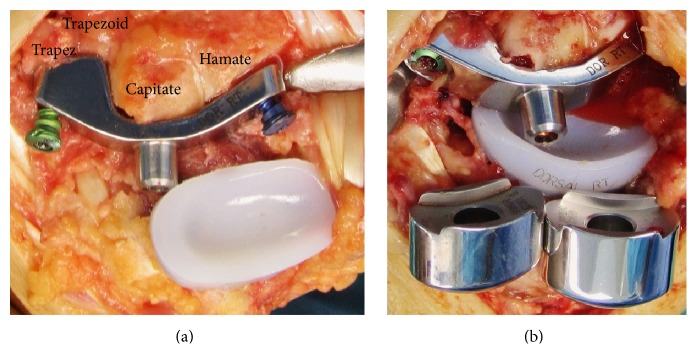
Technical note (Maestro WRS): (a) clinical photo demonstrating fixation of carpal component using a green-colored (mobile head) polyaxial and a blue-colored (rigid head) fixed locking screw; (b) clinical photo demonstrating placement of carpal heads onto the conus of previously inserted carpal component.

Total wrist arthroplasty (TWA) is the motion-preserving alternative to partial or total wrist fusion following posttraumatic wrist joint OA. The noncemented Maestro total wrist (Biomet, Warsaw, Indiana, USA), developed in* 2002* by* Strickland JW (Indiana University, Indianapolis)/Palmer AK (Medical University Syracuse, New York)/Graham TJ (Cleveland Clinic, Ohio)* and available since January 2005, is a third-generation TWA type that is currently in use [12–17]. A further development is the angle-stable Maestro Wrist Reconstructive System (WRS; Biomet, Warsaw, Indiana, USA); theoretically, this type has an advantage in avoiding the inherent risk of carpal component failure by reducing the shear-forces at the implant-to-bone interface using green-colored variable or blue-colored fixed locking screws (Figure 1(a)). The second change is that the intercalated carpal heads are added externally onto the conus of carpal component and not fixated distally over the peg of carpal component as in Maestro total wrist (Figure 1(b)).

It is our hypothesis that TWA using the Maestro WRS can provide satisfactory results in terms of range of motion, pain, and function for immediate salvage of a highly comminuted intra-articular fracture that is not amenable to open reduction and internal fixation (ORIF).

## 2. Case Reports

### 2.1. Case  1

**Figure 2:**
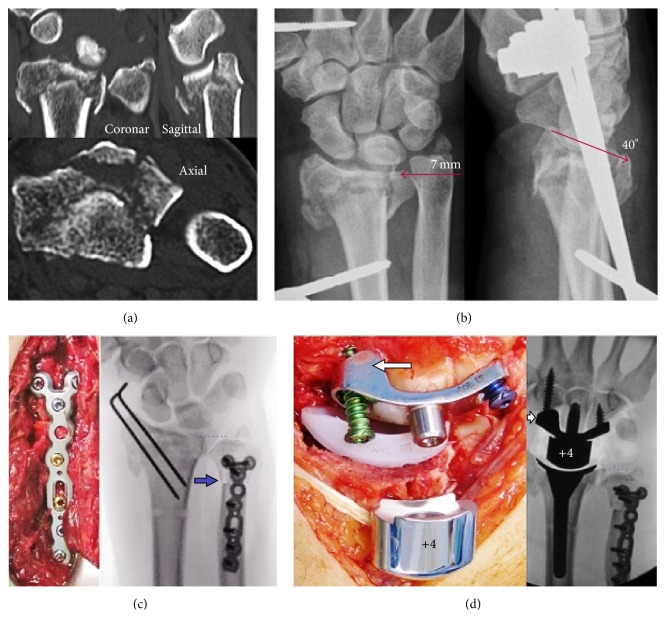
Case report 1 (preoperative and surgical procedures): (a) initial CT images in three levels; (b) AP and lateral radiographs after closed reduction and external fixation; (c) intraoperative clinical photo and AP fluoroscopy after removal of external fixateur, percutaneous pinning, and USO (*arrow*) showing no ulnar-positive variance (*points*); note the displaced ulnar styloid base fracture; (d) intraoperative clinical photo and AP fluoroscopy after Maestro WRS insertion showing correct position of implant; note the scaphoid augment of the carpal component after complete scaphoid excision (*arrow*).

A 56-year-old right-handed male patient with adequate bone stock presented with a highly comminuted intra-articular DRF right after a high-energy fall from a height of three meters (Figure 2(a)). There was no history of any additional trauma or distal radioulnar joint (DRUJ) OA. After closed reduction and external fixation (CREF), the anterior-posterior (AP) and lateral radiographs showed unchanged multiplanar displacement with complete DRUJ incongruity due to a pronounced radial shortening of 7 mm, severely destroyed radial articular surface, and excessive substantial dorsal tilt of 40° (Figure 2(b)). The patient expressed a desire to resume his work in a fast, pain-free, and motion-preserving manner; he works as a tiler in his own company and without any employees. Thus, the shortening of the forearm by using a distal diaphyseal ulnar shortening osteotomy (USO) combined with a TWA was indicated. Two weeks after injury, the external fixateur was removed to avoid pin track infection; the fracture was stabilized additionally with two percutaneously drilled K-wires, and the metaphyseal USO using an angle-stable 2,5 mm multidirectional TriLock titanium APTUS plate (Medartis, Basel, Switzerland) was performed. Intraoperatively, there was no evidence of ulnar-positive variance (Figure 2(c)). Four weeks after injury, the noncemented angle-stable Maestro WRS was inserted (Figure 2(d)). After surgery, the right forearm was immobilized in a cast for one week. Strengthening was started after the sixth postoperative week. Thirteen weeks after injury, the patient returned to full duty at work by using a daily wrist orthosis to minimize the risk of implant luxation by his hard work.

**Figure 3:**
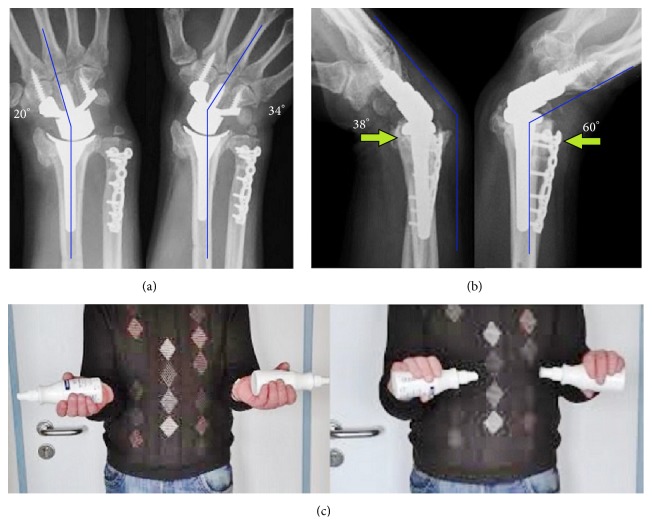
Case report 1 (1-year follow-up): (a) AP radiographs with terminal range of motion: no signs of loosening of either implant and no impingement or impaction; note the union of USO; (b) lateral radiographs with terminal range of motion: no subluxation of distal ulna (*arrows*); (c) clinical photo with supination-pronation arc on both sides.

At the 1-year follow-up, there was no change in position of both implants without any signs of loosening, no impingement, a complete union of USO, and no subluxation of the distal ulna (Figures 3(a)-3(b)). The wrist extension (60°)-flexion (38°) arc was 85,2% of the opposite wrist. The wrist radial deviation (20°)-ulnar deviation (34°) arc was 60,9% of the opposite wrist. The forearm supination (90°)-pronation (80°) arc was 94,4% of the opposite wrist (Figure 3(c)). The grip strength was 85,3% of the opposite wrist with 11 kgf (Jamar dynamometer). The Disabilities of the Arm, Shoulder, and Hand (DASH) score was 17 and the pain 1 point on the visual analog scale (VAS, scale 0–10). The patient reported that he would have the same procedures again. The removal of APTUS plate at distal ulna is not desired by the patient.

### 2.2. Case  2

**Figure 4:**
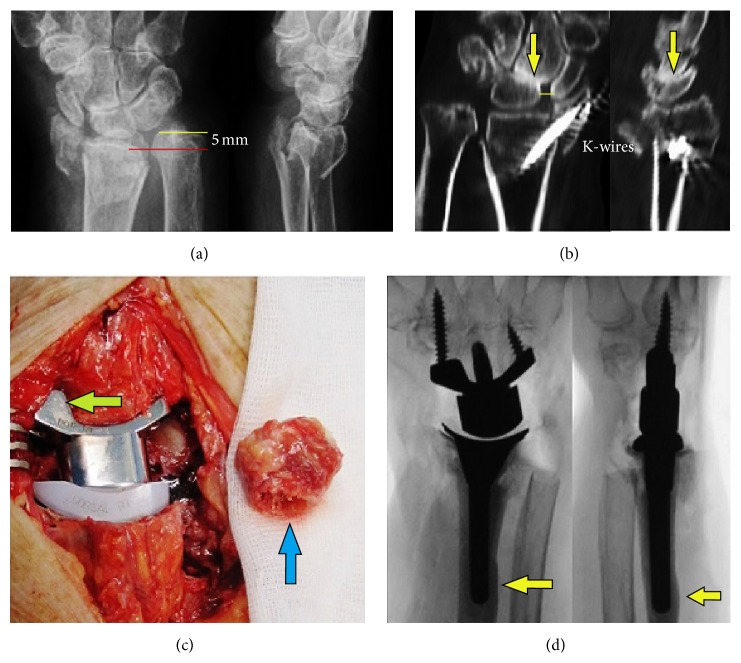
Case report 2 (preoperative and surgical procedures): (a) initial AP and lateral radiographs; (b) coronary and sagittal CT images after removal of external fixateur and percutaneous pinning demonstrating a step-off in the radial articular surface of 3 mm and widened scapholunate gap (*line*) with midcarpal OA (*arrow*); (c) intraoperative clinical photo after Maestro WRS insertion by using a scaphoid augment of the carpal component after complete scaphoid excision (*arrow*) and resected ulnar head (*arrow*); (d) intraoperative AP and lateral fluoroscopy showing correct position of implant and the leakage of cement from one fixateur pin-hole (*arrows*).

An 84-year-old right-handed female patient with poor osteoporotic bone stock presented with a highly comminuted intra-articular DRF right including radial shortening of 5** **mm after a low-energy fall on the ground floor (Figure 4(a)). There was a history of one additional trauma in her right wrist many years ago; however, subjectively it had healed without pronounced impairment of wrist joint motion. After CREF, unchanged multiplanar displacement was present. The shortening of the forearm by a TWA combined with an ulnar head resection (*Darrach* procedure) was indicated. Two weeks after injury, the external fixateur was removed to avoid pin track infection; the fracture was stabilized additionally with two percutaneously drilled K-wires. After that, the computed tomography (CT) images showed severely destroyed radial metaphysis with radial translation, a step-off in the articular surface of 3** **mm, and preexisting SLAC II (Figure 4(b)). Four weeks after injury, insertion of the angle-stable Maestro WRS with cementing of radial component combined with ulnar head resection was performed (Figure 4(c)). Some cement leaked through one fixateur pin-hole at the distal radial diaphysis (Figure 4(d)); however, this did not cause functional irritation of the interosseous membrane. After surgery, the right forearm was immobilized in a cast for one week. Strengthening was started after the sixth postoperative week. This widowed female patient cares for her 54-year-old paralyzed daughter at home, who has been suffering from multiple sclerosis for many years.

**Figure 5:**
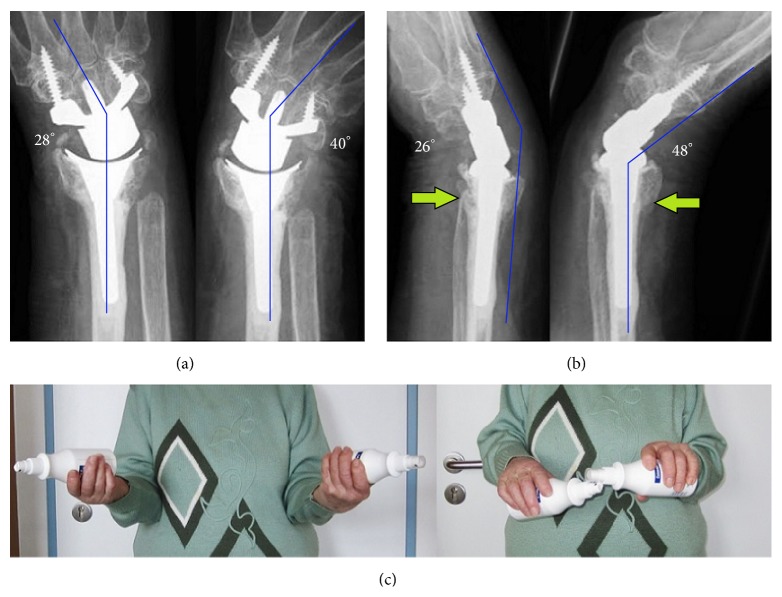
Case report 2 (1-year follow-up): (a) AP radiographs with terminal range of motion: no signs of loosening of either implant and no radioulnar impingement; (b) lateral radiographs with terminal range of motion: no subluxation of distal ulna stump (*arrows*); (c) clinical photo with supination-pronation arc on both sides.

At the 1-year follow-up, there was no change in position of both implants without any signs of loosening, no impingement, and no subluxation of the distal ulna (Figures 5(a)-5(b)). The wrist extension (48°)-flexion (26°) arc was 77,9% of the opposite wrist. The wrist radial deviation (26°)-ulnar deviation (40°) arc was 97,1% of the opposite wrist. The forearm supination (90°)-pronation (80°) arc was 94,4% of the opposite wrist (Figure 5(c)). The grip strength was 77,8% of the opposite wrist with 7 kgf (Jamar dynamometer). The DASH score was 27 and the pain 2 points on VAS. The patient reported that she would have the same procedures again.

## 3. Discussion

Highly comminuted DRFs represent a challenging therapeutic problem. The early pain-free and sufficient wrist motion in elderly patients with DRF is mandatory to achieve their independence in quality of life; an inevitable immobilization for several weeks leads to reduction in range of motion, deterioration of muscle strength, and malfunctions of fine motor skills as well as changes of motor and sensory representations in the brain [18]. In patients aged 65 years and older, the averaged incidence of malunion for all surgical procedures (ORIF, CREF, and pinning) is reported to be 29% [19]. The primary shortening of the forearm for treatment of severely comminuted distal forearm fractures using the* Sauvé-Kapandji* arthrodesis or* Darrach* procedure is also an option for elderly osteoporotic patients if the radial articular surface can be restored by volar plating and may help avoid secondary procedures related to posttraumatic DRUJ OA or ulnar impaction syndrome [20–22]. The ulnar head replacement (UHR) would be the salvage option in our* case 2* if an intrinsically unstable construct occurs [15, 23, 24].

The total or partial replacement has proven to be a suitable and reliable treatment option for selected elderly patients with highly comminuted fractures in the shoulder, elbow, and knee joint [25–28]. It is comparable to complex wrist joint fractures; autonomous elderly patients with AO type “C 2/3” are good candidates to primary prosthetic surgery. The radial resurfing hemiarthroplasty with or without replacement of distal radius metaphysis using the Sophia (Biotech, Paris, Frankreich) implant [8, 9], the radial component of RE-MOTION (Small Bone Innovations, Morrisville, Pennsylvania, USA) total wrist [10], the “Cobra” (Groupe Lépine, Lyon, France) implant [10], and the “Prosthelast” (Argomedical, Cham, Switzerland) implant [11] offers a useful alternative to other procedures and may help avoid secondary procedures related to posttraumatic wrist joint and DRUJ OA. Radial hemiwrist implants may also help avoid the main problem of carpal component failure in TWA. Wrist replacement may be performed before or after shoulder or elbow surgery but prior to hand surgery to improve hand balance and optimize rehabilitation of digits [29].

First results with the Maestro total wrist in 2009 have been encouraging; a series of 19 patients with an average follow-up of 27 months revealed no prosthetic loosening, satisfactory pain relief, and an average DASH score of 22 [12]. A second study in 2012 revealed no evidence of radiological prosthetic loosening or subsidence in 22 patients (23 wrists, average age 63 years ranging from 49 to 79 years) at an average follow-up of 28 months (DASH score 31, VAS 2) and showed statistically significant improvement of radial deviation; in detail, complications were four cases of wrist contracture, one case of prosthetic failure resulting from deep infection, one case of synovitis, and one case of instability [13]. A third study in 2013 at a 56-month follow-up (*N* = 7, average age 64 years, ranging from 60 to 77 years) did not reveal radiological loosening or osteolysis; the outcome was statistically rated significantly better at 31 by the patients using the Patient Related Wrist Evaluation (PRWE) compared to 73 in patients who had undergone total wrist fusion (*N* = 15), respectively [14]. A fourth publication in 2014 (case report, 55-year-old man) at a 5-year follow-up showed asymptomatic radiolucency at the tip of radial stem and local bone resorption under the offset of radial component within the first and second postoperative years but without progression in the further course and no evident radial impingement with terminal active radial deviation [15]. The most favorable functional outcome of Maestro total wrist for radial-to-ulnar deviation and extension in combination with the high patient satisfaction using Canadian Occupational Performance Measure (COPM) compared to other third generation types, published in a fifth study with a large series of 62 patients (average age 59 years ± 12,5) in 2015, may be justified in preserving resection-related carpal height due to its three various carpal heads in combination with its design of ellipsoid surface articulation [16]. Using the RE-MOTION total wrist, the reason for its limited radial deviation with radial impingement between the carpus and radial styloid or radial prosthesis component which had also been demonstrated radiologically in one patient with terminal active radial deviation [7] appears to be caused by its resection-related reduced carpal height [16].

We present preliminary experience regarding the relatively new angle-stable Maestro WRS. Currently, it cannot be said whether the angle-stable fixation is able to solve the main problem of carpal component failure; however, the biomechanical advantage using angle-stable locking plates at the distal radius especially in osteoporotic bone stock is well-known [30]. The cemented radial insertion of third TWA generation types (as in our* case 2*) is also recommended as salvage option for insertion of the RE-MOTION total wrist if the bone stock is poor [31].

The diaphyseal or metaphyseal USO is the treatment option in patients without osteoporosis, with no carpal malalignment, and with no preexisting evident DRUJ OA as in* case 1* [32–34]. Using the USO following malunited distal radius fractures, the substantial volar or dorsal tilt should be less than 20° [32]. However, the diaphyseal USO has been consistently beset with complications such as irritation from hardware, tendonitis, delayed or nonunion, refracture, and DRUJ incongruity [33, 34]. The metaphyseal USO is limited by a wafer resection distance up to 5 mm [33]. One study has shown that there is a significantly increased rate of DRUJ OA in patients who had undergone USO following malunited DRF at a 7-year follow-up [35]. For failed USO or posttraumatic DRUJ OA, ulnar head hemiresection procedures (*Bowers*,* Watson*), an UHR, and finally the* Darrach* procedure continue to be the salvage options for this patient [15, 23, 24].

It is not the intention of our presented two case reports in a short-term follow-up to advocate for general use of TWA in treatment of highly comminuted DRF. Currently, it must be emphasized that this procedure should be considered for selected older and elderly patients only who need a fast and pain-free restoration of their ability to work and independence in their personal, professional, and social environment. Radiologically, in both patients there were unacceptable criteria after primary surgical procedures potentially leading to poor functional outcome and patient's disability, posttraumatic wrist joint OA, and required revision procedures in the further course resulting in much more prolonged time of inability to work. Hard physical occupations as in our* case 1* are not generally considered a contraindication for TWA [36]. Both Maestro types have advantages in design and functional outcome, but also disadvantages in comparison with other third generation TWA types. The weak point is that currently there are no published reliable long-term results with the Maestro total wrist regarding its cumulative survival rate. The convincing functional outcome in our both patients cannot be compared with other studies on TWA because there was no history of long-standing impaired wrist joint motion before injury. Additional surgical procedures regarding concomitant DRUJ injury in highly comminuted DRF must also be focused on special features such as patient's age, bone stock, physical demand, and reliable salvage options individually. Further experience is needed to validate this concept.
